# Positional isomerism and short hydrogen bonds govern photochromic behaviour in dipyridyl-NDI⋯formic acid co-crystals†

**DOI:** 10.1039/d6ce00262e

**Published:** 2026-05-29

**Authors:** Adnan Ishaq, Louise Male, Neil R. Champness

**Affiliations:** a School of Chemistry, University of Birmingham Edgbaston Birmingham B15 2TT UK n.champness@bham.ac.uk

## Abstract

Positional isomerism in pyridyl naphthalene diimide formic acid co-crystals controls crystal symmetry, yielding centrosymmetric (*I*2/*a*) and polar (*Pc*) structures from 4- and 2-pyridyl isomers respectively. Both materials function as reversible photochromic switches upon UV irradiation *via* a proton-coupled electron transfer type mechanism, generating NDI radical anions, as confirmed by UV-vis and ATR-FTIR spectroscopy.

## Abstract 2

Positional isomerism in pyridyl naphthalene diimide formic acid co-crystals controls reversible photochromic switching behaviour upon UV irradiation *via* a proton-coupled electron transfer type mechanism.

## Graphical abstract

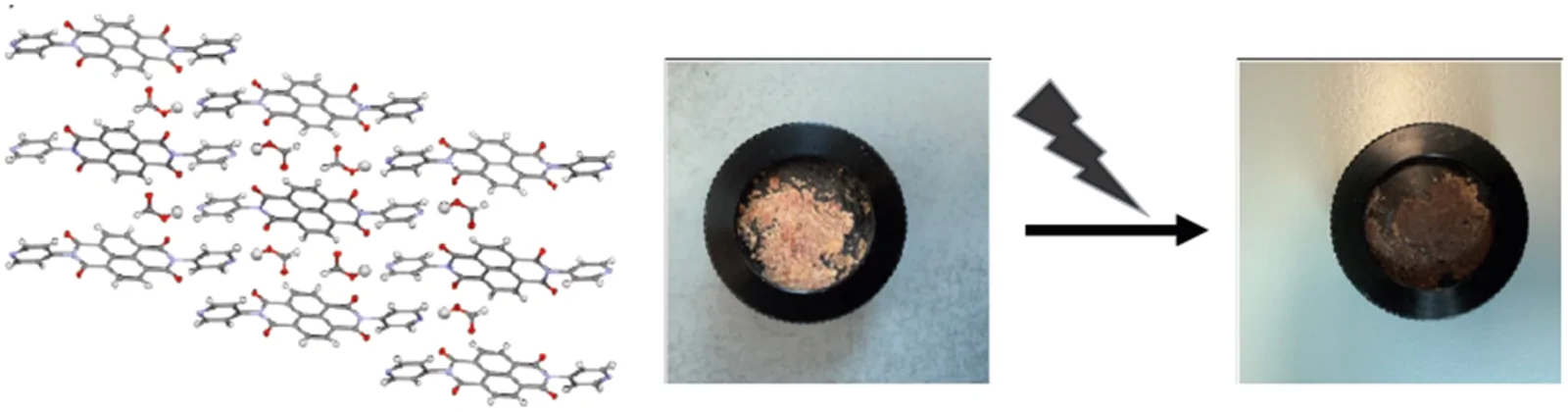

Naphthalene diimides (NDIs) are a well-established family of electron-deficient aromatic compounds which are widely employed in supramolecular and radical chemistry.^1–5^ Due to the presence of their large and rigid aromatic core which exhibits a strong inclination towards π–π stacking, in combination with the synthetically accessible peripheral substitution through their imide positions, NDIs are often employed in the construction of a large range of crystalline materials including metal–organic frameworks (MOFs), hydrogen-bonded organic frameworks (HOFs) and molecular co-crystals.^6–8^

NDIs have a low-lying LUMO which renders them readily reduced to form the well-known NDI radical anion (NDI˙^−^).^9^ This is formed by a single-electron reduction of the aromatic core and is among the most well-characterised organic radical species in the literature, often exhibiting characteristic UV-vis absorptions in the 600–800 nm region.^10,11^ In the solid state more specifically, photochromic behaviour of NDI radicals is an area of growing interest, and such behaviour has been reported across a range of systems such as MOFs, coordination polymers, HOFs and crystalline hybrid materials.^7,12–15^ The exact nature of the electron donor in a specific system can be difficult to establish and the exact structural factors which govern the efficiency and reversibility of the radical formation remain poorly understood in the solid-state. Therefore, simpler NDI-donor co-crystal systems represent an emerging platform for mechanistic insight of such processes.^16^

Co-crystals of hydrogen-bond acceptor substituted NDIs with hydrogen-bond donors offer a direct route to producing photochromic materials. The hydrogen-bonding interface allows for the modification of the photochromic properties through a crystal-engineering approach by the tuning of the donor–acceptor geometry.^17,18^ NDI derivatives bearing pyridyl groups at the imide positions are attractive in this context as this functional group facilitates a relatively simple co-crystallisation with common acids. The Δp*K*_a_ framework outlined by Cruz-Cabeza *et al.* can be used to predict whether the outcome will be a co-crystal or salt and additionally the nature of the hydrogen-bond produced between the two coformers.^19^ More specifically, careful consideration of the Δp*K*_a_ between the pyridyl unit and the carboxylic acid can yield low-barrier hydrogen bonds which can facilitate proton transfer and may serve to activate the carboxylic acid coformer to serving as an electron donor to the NDI.^20^

In this work we investigate the effect of positional isomerism of the pyridyl-NDI units on the overall crystal packing by co-crystallising 2- and 4-pyridyl substituted NDIs with formic acid. An approach which retains molecular composition while altering potential hydrogen-bonding vectors. As such, we observe a striking difference in crystal symmetry between the two with D4PNDI·2FAH yielding a centrosymmetric structure (*I*2/*a*), while D2PNDI·3FAH forms a polar crystal structure (*Pc*) through the adoption of a *syn*-type conformation of the pyridyl groups in the NDI. We observe strong hydrogen-bonding between formic acid and the pyridyl groups in both structures through short O–H⋯N contacts consistent with partial proton transfer, while C–O bond length analysis supports a co-crystal assignment to these systems rather than them being simple salts. In addition, we observe a photochromic response in both materials upon irradiation with 365 nm UV light as NDI˙^−^ is formed. We report photophysical characterisation and FTIR spectroscopy supporting a proton-coupled electron transfer (PCET) type mechanism which should be enhanced by the low-barrier hydrogen-bonds.^21,22^ With that, we suggest rationale which links their contrasting photophysical behaviour to their crystal structures.

To investigate the effect of pyridyl substitution patterns on the overall crystal structure and packing of pyridyl NDI co-crystals with formic acid, we synthesised a series of dipyridyl NDI derivatives (2- (D2PNDI), 3- (D3PNDI) and 4-pyridyl (D4PNDI)). These species were crystallised by dissolution in neat formic acid followed by slow evaporation. Large, high-quality crystals were obtained for the 2- and 4-isomers over the course of 24 hours. Attempts to obtain crystals of quality for SCXRD analysis of the 3-isomer were unsuccessful, including experiments employing a pin-holed vessel to reduce the rate of formic acid evaporation.

D4PNDI crystallises with two formic acid molecules per NDI molecule, with half of an NDI and one formic acid per asymmetric unit (Fig. 1a). Crystals of D4PNDI·2FAH form in the monoclinic space group *I*2/*a* (Table S1) and forms an assembly with channels, propagating into the crystallographic *b* direction, in which are located formic acid molecules sitting between π–π stacked NDI species (Fig. 1b). Notably, the formic acid molecules act as hydrogen bond donors to the pyridyl nitrogen atoms at either end of the NDI forming strong hydrogen-bonds (O101⋯N1 = 2.631(2) Å). This is just longer than the accepted O–H⋯N cutoff of 2.6 Å below which these are formally considered short strong hydrogen-bonds (SSHBs).^23^ Nevertheless, with such a short hydrogen-bond, a low barrier for proton transfer between the carboxylic acid and pyridyl units would be anticipated.

**Fig. 1:**
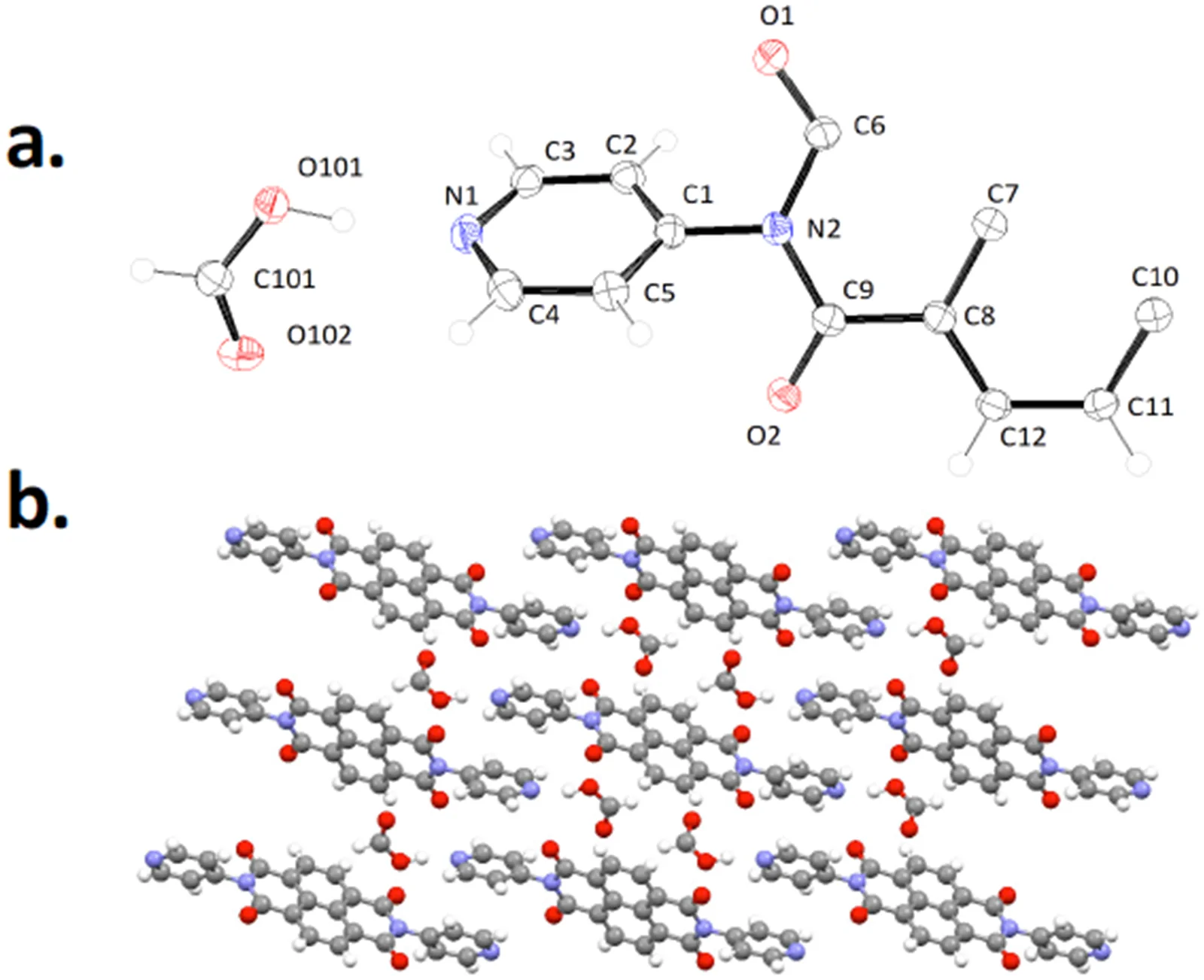
a) ORTEP diagram showing the asymmetric unit of D4PNDI·2FAH with key atoms labelled. Displacement ellipsoid are drawn at the 50% probability level. b) Packing view along the *b* axis showing the FAH containing channels between NDI molecules.

No hydrogen bonds are observed between adjacent formic acid molecules within the channels. This indicates that the acid units act solely as caps for the NDI units rather than forming self-associated hydrogen-bonded networks. Parallel to the formic acid channels are slightly offset π–π stacking interactions between the NDI cores running along the same axis with separation of approximately 3.5 Å (Fig. S1). Orthogonally, along the crystallographic *c* axis, more direct π–π stacking is observed between pyridyl units with a separation of around 3.6 Å (Fig. S1), these values are consistent with those found in the literature.^24,25^

From the crystal structure we observe that the formic acid oxygens have inequivalent C–O bond lengths (C101–O101 = 1.31 Å, C101–O102 = 1.21 Å) (Fig. S2) suggesting the presence of an OH group, rather than a delocalised carboxylate group, and therefore that the proton remains associated with the formic acid molecule rather than protonating the pyridyl nitrogen. In aqueous solution, formic acid (p*K*_a_ ≈ 3.75) and pyridinium (p*K*_a_ ≈ 5.25) give only a modest Δp*K*_a_ ≈ 1.5 which places this acid–base pair in the intermediate regime (Δp*K*_a_ = −1 to 4), where either salt or co-crystal formation can occur as described by Cruz-Cabeza *et al.*^19,26–28^ The formation of a co-crystal in this case, rather than a salt, may be promoted by the use of glacial formic acid rather than *via* an acidic aqueous medium – which is known to stabilise ionic species.^29,30^ In addition to this, Ilgen *et al.* demonstrate that the p*K*_a_ for formic acid increases under nanoconfinement.^31^ Given that the structure contains formic acid present in channels confined by NDI molecules, a decreased value for Δp*K*_a_ stemming from this could potentially lead to observation of a co-crystal rather than the corresponding salt.

In contrast to D4PNDI, D2PNDI crystallises with three formic acid molecules per NDI molecule in its corresponding co-crystal. D2PNDI·3FAH adopts the monoclinic, non-centrosymmetric space group *Pc* (Table S1). The asymmetric unit comprises of four D2PNDI molecules each with three formic acid molecules, which assemble to form a discrete hydrogen-bonded trimer, for a total of four NDI and twelve formic acid molecules per asymmetric unit (Fig. S3). Within the trimeric acid motif, the two outermost formic acid species donate strong O–H⋯N hydrogen bonds (O–H⋯N ≈ 2.6 Å) to the pyridyl nitrogen atoms of the NDI. The third formic acid molecule participates in an O–H⋯O hydrogen bond with one of the neighbouring acids completing the trimeric assembly (Fig. 2a). Notably, both pyridyl nitrogen atoms of each NDI point in the same direction, in a *syn*-orientation (Fig. S4), such that both hydrogen bond acceptor sites are on the same side of the NDI molecule, relative to the planar core.

**Fig. 2:**
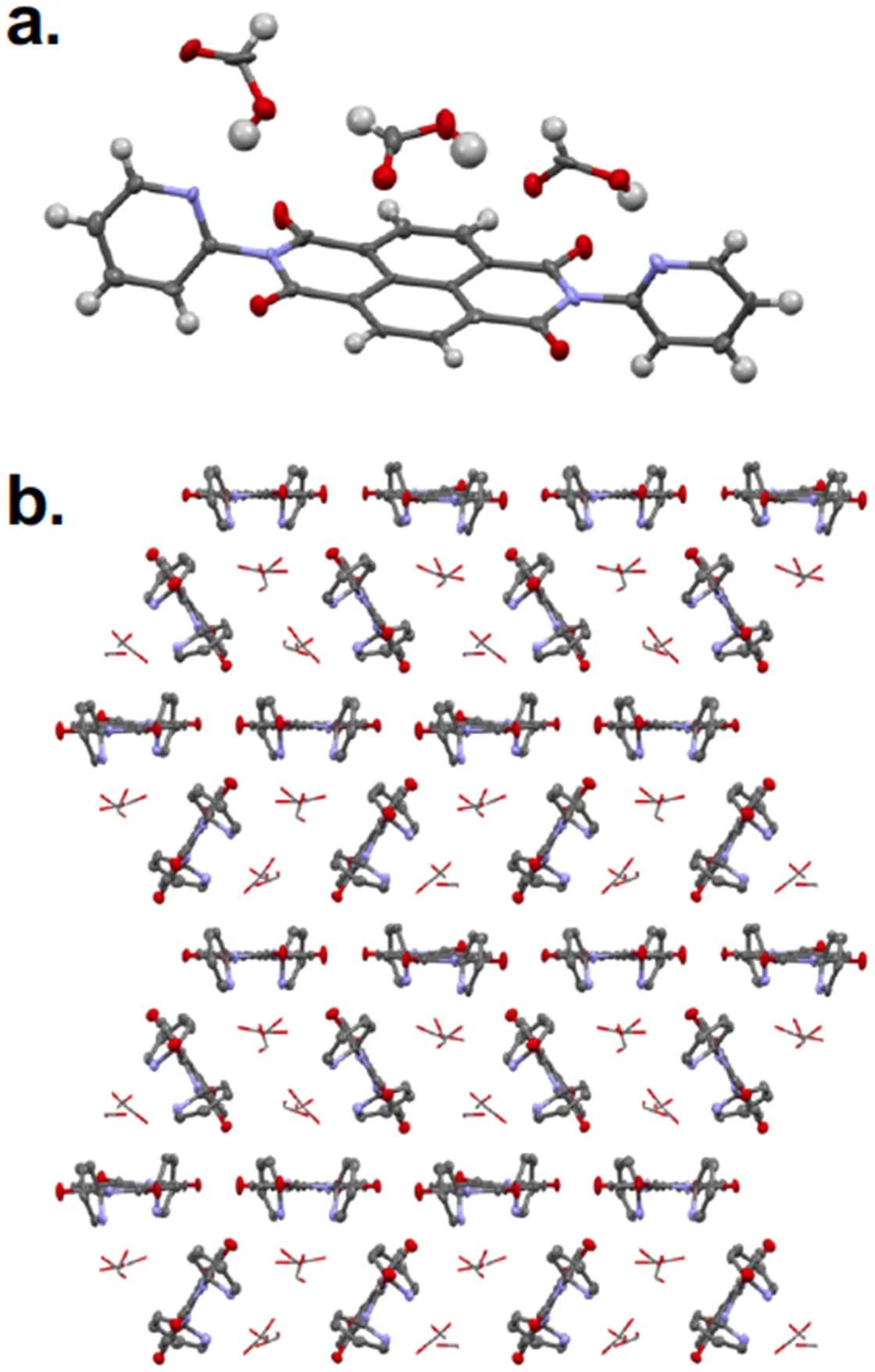
a) The *syn*-arrangement of pyridyl groups in D2PNDI which engages the FAH trimer. b) Channels defined by NDI molecules, hydrogen atoms are omitted for clarity.

Extension of the NDI_4_(FA)_12_ motif through the *c*-glide symmetry operation, and translational symmetry yields two laterally related orientations of the NDI molecules (relative to the *b* axis). Adjacent layers display alternating orientations of both lateral pyridyl groups. In all cases of the axial NDI molecules which lie along the *bc* plane, both pyridyl nitrogen atoms are directed towards the same side of the NDI core, resulting in a net polar crystal structure.

At a packing level, this leads to the formation of infinite one-dimensional channels extending parallel to the crystallographic *a* axis (Fig. 2b). These are defined by the NDI cores, which are aligned parallel to the FAH trimers. The channels have alternating oblique cross sections as a result of the alternating arrangement of lateral NDI molecules. While the NDI molecules which make up the “side walls” of the channels alternate between having both nitrogen atoms pointing left, and both to the right, all NDI molecules which make up the “top and bottom walls” have their nitrogen atoms pointing the same direction along the crystallographic *c* axis, giving rise to a polar structure (Fig. S5). The directional anisotropy of the pyridyl nitrogen atoms is consistent with structure solution in the crystallographically polar space group *Pc*.

As observed for D4PNDI·2FAH, D2PNDI·3FAH is best described as a co-crystal rather than a salt, with the protons remaining associated with the formic acid molecules rather than forming pyridinium species. This is supported by inequivalent C–O bond lengths for all formic acid species present in the structure.

One could imagine a structure where the *anti*-conformation of D2PNDI could lead to a vastly different, centrosymmetric outcome as a result of the presence of an inversion symmetry element at the centre of the NDI molecule. In this case however, we observe a non-centrosymmetric structure due to the presence of the *syn*-conformation templated by the formic acid trimers, this result may provide future direction into producing further polar co-crystals by promoting the *syn*-conformation in the solid-state (Fig. S5).

It was found that both D4PNDI·2FAH and D2PNDI·3FAH undergo a colour change upon irradiation by 365 nm UV light. Upon irradiation D4PNDI·2FAH quickly turns from red to dark brown while D2PNDI·3FAH turns from white to red-brown (Fig. 3 and S6). No such response was observed for the parent D4PNDI or D2PNDI species, suggesting that in this case, the FA within the co-crystals acts as an electron donor to form NDI˙^−^. The colour of both co-crystals returns to the ground state after approximately two hours.

**Fig. 3:**
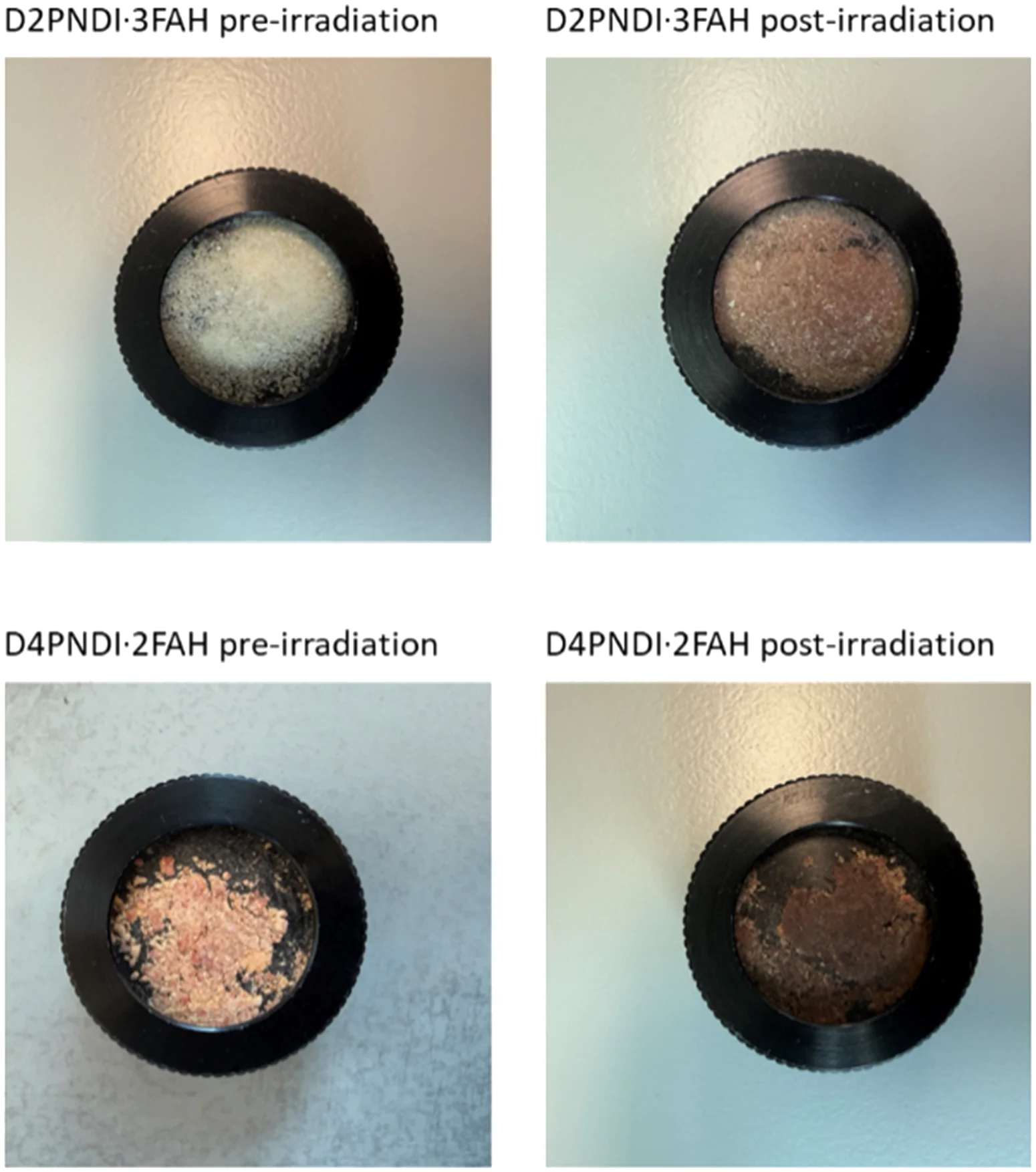
Diffuse reflectance UV-vis sample holders containing powdered D4PNDI·2FAH and D2PNDI·3FAH demonstrating the colour change observed upon irradiation by 365 nm.

Diffuse reflectance UV-vis spectroscopy was performed on powdered samples of irradiated D4PNDI·2FAH and D2PNDI·3FAH and collected at 40 second intervals up to 4000 seconds post-irradiation using an integrating sphere and BaSO_4_ as a reference. The results show a significantly different photochromic behaviour between the two structures (Fig. 4). Upon irradiation by 365 nm UV for 120 seconds, the UV-vis spectra of D4PNDI·2FAH reveals the growth of a large broad underlying absorption plus a distinct absorption band at approximately 640 nm. This is assigned to the formation of NDI˙^−^ based on extensive literature precedent.^7,10,13^ The response in D2PNDI·3FAH is far weaker suggesting that the formation of the radical is less efficient in this system however a new, weak, absorption band is observed at 770 nm. Analysis of the spectra shows the presence of absorptions beginning above 800 nm which may be attributed to interactions between NDI radicals helped by π–π stacking (NDI⋯NDI = 3.48 Å) in the D4PNDI crystal structure.^32,33^ Additionally, intervalence charge transfer between an NDI radical and a neutral NDI molecule may explain this feature.^34^ The absence of this absorption can be attributed to the lack of this important π–π interaction between NDI units in the D2PNDI structure.

**Fig. 4:**
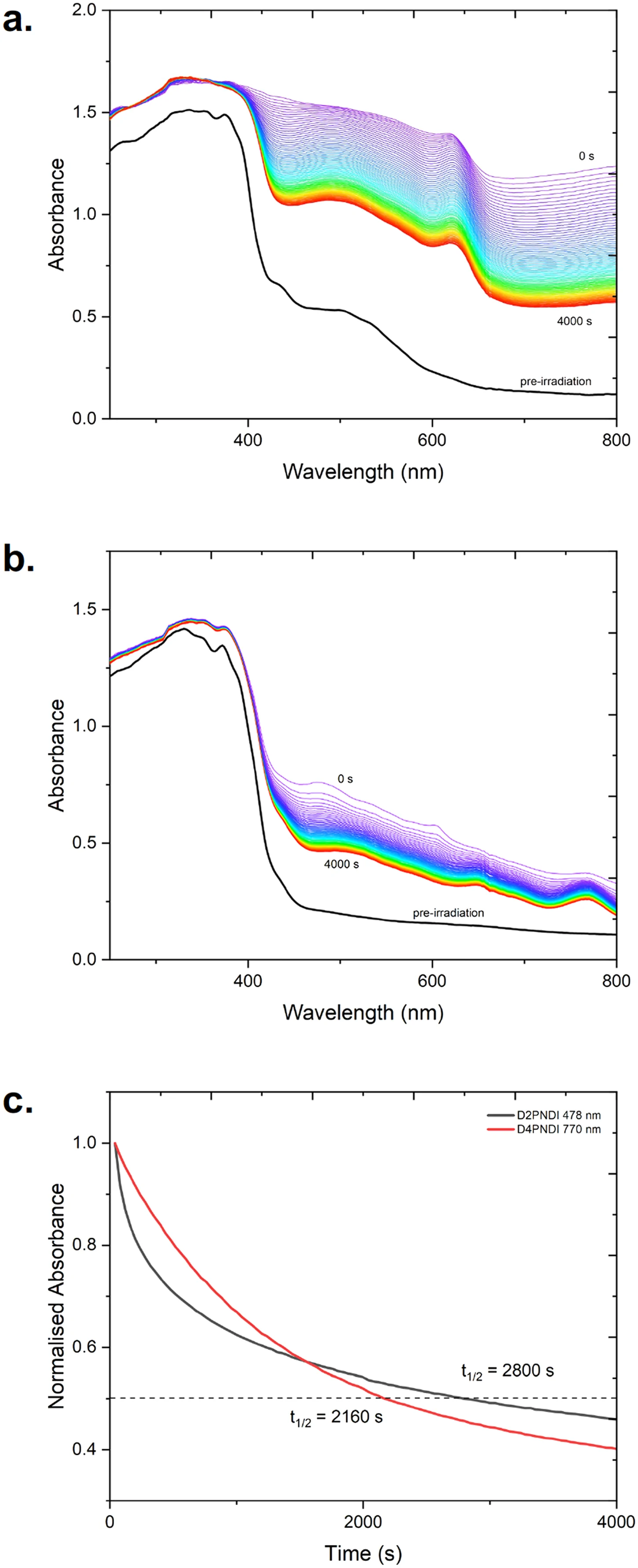
UV-vis spectra of a) D4PNDI·2FAH and b) D2PNDI·3FAH collected over 4000 seconds post irradiation by 365 nm UV; c) absorbance over time of selected wavelengths for each crystal structure, used to determine the excited state half-life.

Analysis of the half-lives of the excited state in both co-crystals, by plotting the normalised absorbance at selected wavelengths for the two structures (where the largest change over time is observed), revealed that NDI˙^−^ in the polar D2PNDI·3FAH structure has a significantly longer half-life at approximately 2800 seconds, with the centrosymmetric D4PNDI·2FAH at around 2160 seconds (Fig. 4c). This suggests that a larger energy barrier is present for both the ground state to become the excited state, and also for the relaxation back to the ground state.

Given the presence of short hydrogen bonds in both systems, a proton-coupled electron transfer (PCET) mechanism was considered. The short O⋯N contacts suggest partial proton transfer character at the hydrogen bond, and formate formed upon full proton transfer to the pyridine nitrogen would be a considerably stronger electron donor than neutral formic acid and is known to undergo one-electron oxidation to form the formyloxyl radical.^35^ Additionally, photoinduced proton transfer in hydrogen-bonded pyridine–carboxylic acid systems is also documented in the solid state.^36^ In this interpretation, energy provided by the UV irradiation drives proton transfer to form formate and pyridinium, the formate undergoes one-electron oxidation to form a formyloxyl radical with concomitant reduction to form NDI˙^−^ (Fig. 5). This hypothesis was probed by solid-state ATR-FTIR spectroscopy before and after UV irradiation (365 nm, 120 s).

**Fig. 5:**
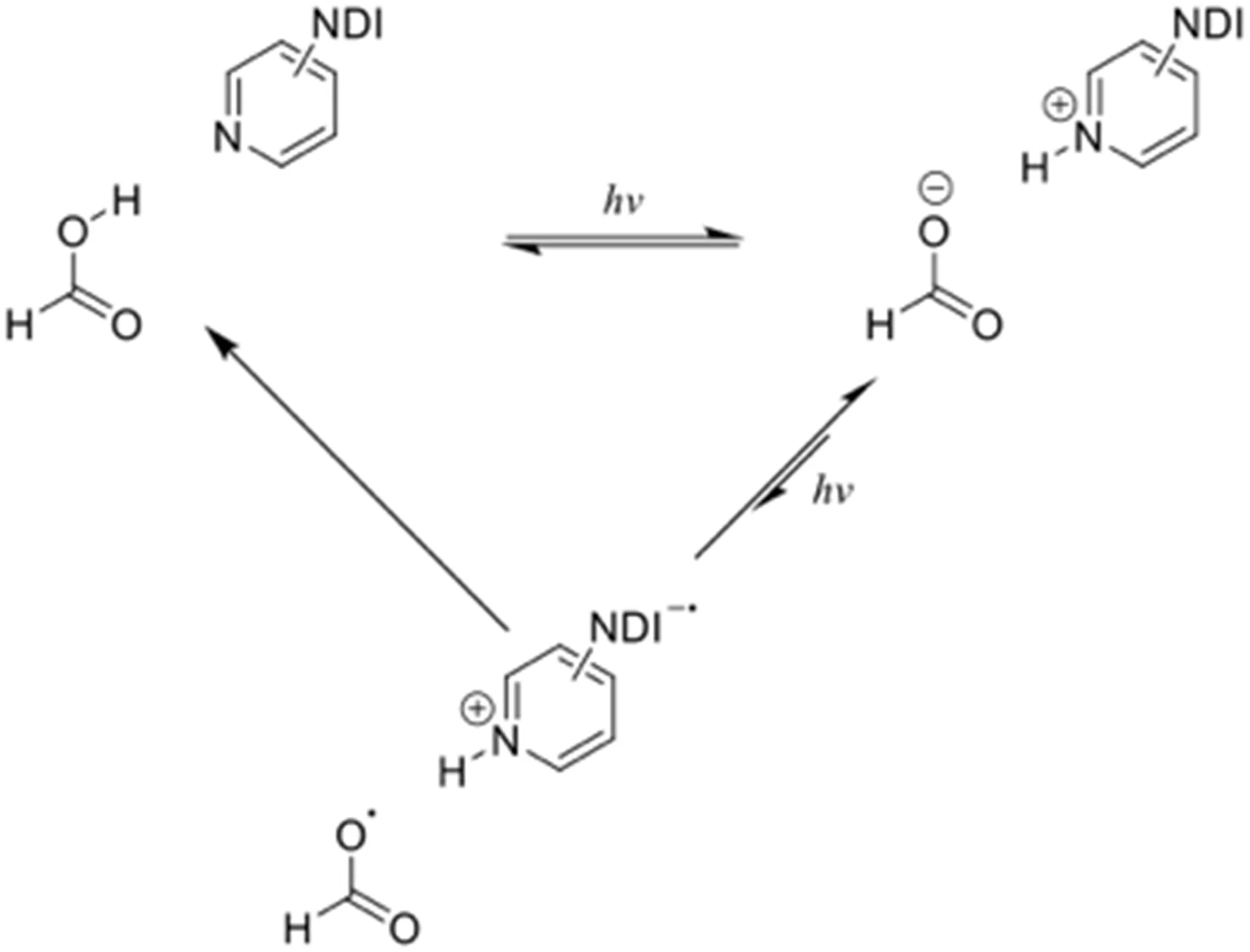
PCET mechanism for the formation of formate and its subsequent one-electron oxidation to form NDI˙^−^.

The ATR-FTIR spectrum of D4PNDI·2FAH pre-irradiation (Fig. S7) shows a prominent C=O stretching band at 1708 cm^−1^ consistent with formic acid. Upon irradiation this band attenuates, and a set of new features grow at 1570 and 1330 cm^−1^, attributed to the symmetric and asymmetric C–O stretching modes of the formate anion.^37,38^ Bands at 1650 and 1510 cm^−1^ are assigned to pyridinium ring stretching modes^39,40^ and the band at 945 cm^−1^ is assigned to the N^+^–H out of plane wagging vibration,^39^ consistent with pyridinium formation. No detectable change was observed for the same experiment on D2PNDI·3FAH which is consistent with its reduced photochromic response.

Following from the proposed PCET type mechanism, we suggest that the radical formation efficiency in the polar structure of D2PNDI·3FAH may be hindered by a combination of factors, when compared to D4PNDI·2FAH. The first is that the pyridyl group of D2PNDI is expected to be less basic because of its closer proximity to the electron withdrawing imide groups of the NDI core, deactivating it to the proton transfer step proposed. Secondly, the additional hydrogen-bond donated by the central FAH of the formic acid trimer serves to withdraw electron density from the generated formate and weakens its electron donating capacity to the NDI core (Fig. S8).^41^ Finally, we suggest that the polar crystal structure has an intrinsic dipole which may raise the energy barrier to forming the charge separated excited state.

In conclusion, we have demonstrated that positional isomerism of the pyridyl substituents in symmetric dipyridyl NDIs is a design handle which can control the crystal packing and symmetry outcomes in hydrogen-bonded co-crystals. More specifically, this alteration of hydrogen-bond vectors but retention of molecular composition, is a handle which can lead to different photophysical properties in the solid state. The *para*-isomer yields a centrosymmetric structure (*I*2/*a*) owing to the presence of an intrinsic inversion centre at the core of the NDI molecule. Meanwhile, the *ortho*-isomer generates a polar structure (*Pc*) due to the adoption of a *syn*-conformation templated by a formic acid trimeric motif. In both structures short O–H⋯N contacts are observed providing a low barrier to proton transfer which is prerequisite for PCET type mechanisms.

We observed that both co-crystals undergo reversible photochromic switching upon UV irradiation, a finding which was subsequently attributed to proton-coupled electron transfer between the formic acid and pyridyl units, forming formate and pyridinium species, which can be detected through ATR-FTIR spectroscopy. The polar structure of D2PNDI·3FAH shows a weakened photochromic response but a longer half-life which we speculate are due to a combination of the intrinsic electric dipole of the crystal, and the presence of an additional O–H⋯O contact in the formic acid trimers which attenuate electron density in the formate electron donor.

These results demonstrate that positional isomerism in NDI co-crystals provides a single design variable which controls crystal symmetry, hydrogen-bond geometry and ultimately photophysical behaviour. This work is part of a broader study examining short hydrogen bonds in NDI co-crystals and demonstrates functional properties in these readily synthesised materials.

## Author contributions

The synthesis of materials and all experimental studies were conducted by A. I. Data for single crystal structures and subsequent refinement were conducted by A. I. and L. M. N. R. C. provided supervision. The project was conceived by A. I. and N. R. C. All authors discussed the results, contributed to and have given approval to the final version of the manuscript.

## Conflicts of interest

There are no conflicts to declare.

## Supplementary Material

CE-028-D6CE00262E-s001

CE-028-D6CE00262E-s002

## Data Availability

Supplementary information (SI): SI contains details of synthesis and crystal growth, details of crystallographic measurements and other characterisation techniques. The X-ray crystallographic coordinates for structures reported in this study have been deposited at the Cambridge Crystallographic Data Centre (CCDC), under deposition numbers 2538522 and 2538311. CCDC 2538522 and 2538311 contain the supplementary crystallographic data for this paper.^42*a*,*b*^
