# Supplementary material for: Positional isomerism and short hydrogen bonds govern photochromic behaviour in dipyridyl-NDI⋯formic acid co-crystals
Source: CrystEngComm. 2026 May 29;28(27):4091–6. doi: 10.1039/d6ce00262e (PMC13587030; doi:10.1039/d6ce00262e)
Supplement: Supplementary file 1 [file CE-028-D6CE00262E-s001.pdf]

Supplementary Information (SI) for CrystEngComm.
This journal is © The Royal Society of Chemistry 2026

Supporting Information for:

Positional Isomerism and Short Hydrogen Bonds Govern Photochromic Behaviour in dipyridyl-NDI…Formic Acid Co-crystals

Adnan Ishaq, Louise Male and Neil R. Champness

S1 Synthesis Page 2

S2 Single crystal X-ray Diffraction and additional figures Page 5

S3 Spectroscopic Technique Experimental Page 9

S5 References Page 9

Experimental

Materials

2-aminopyridine, 4-aminopyridine, 1,4,5,8-Naphthalenetetracarboxylic dianhydride (NTCDA) and formic acid (FA) and were all purchased from either Sigma Aldrich, Alpha Aesar or Fluorochem and were used without further purification.

Synthesis of pyridyl naphthalene diimides (NDIs)

All syntheses of the pyridyl NDIs followed a slightly modified literature procedure.[S1] To a 50 mL round-bottom flask containing 20 mL DMF, the amine component (7.5 mmol, 700 mg) was added and this was heated to 100 °C with stirring until completely dissolved giving rise to an orange-brown solution. To this solution NTCDA (3 mmol, 804 mg) was added and heated to 140 °C. The solution turns from a beige suspension to a brown solution as the products react. After 12 hours, the reaction was allowed to cool very slowly to room temperature yielding red/orange crystals for D4PNDI, and off-white crystalline material for D2PNDI. The solid components were then collected by vacuum filtration and washed with cold DMF (3x 5 mL), acetone (3 x 5 mL) and diethyl ether (3 x 5 mL). The products were then collected and dried for 24 hours in a vacuum desiccator before use.

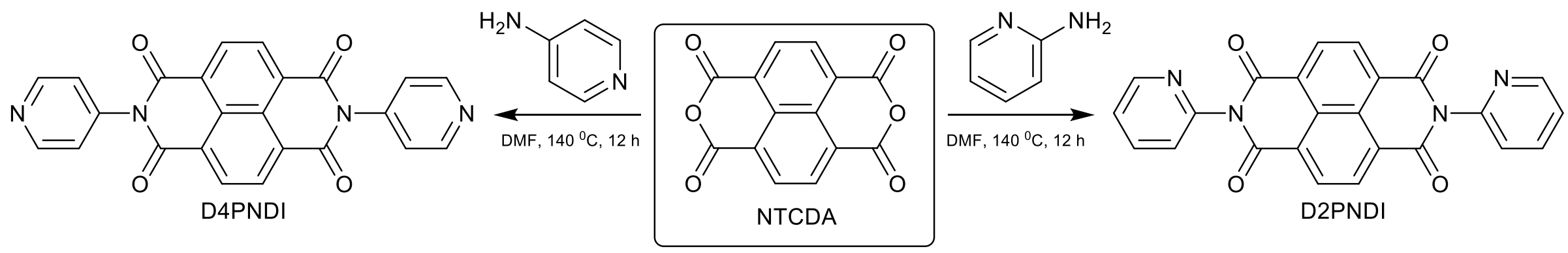

D2PNDI

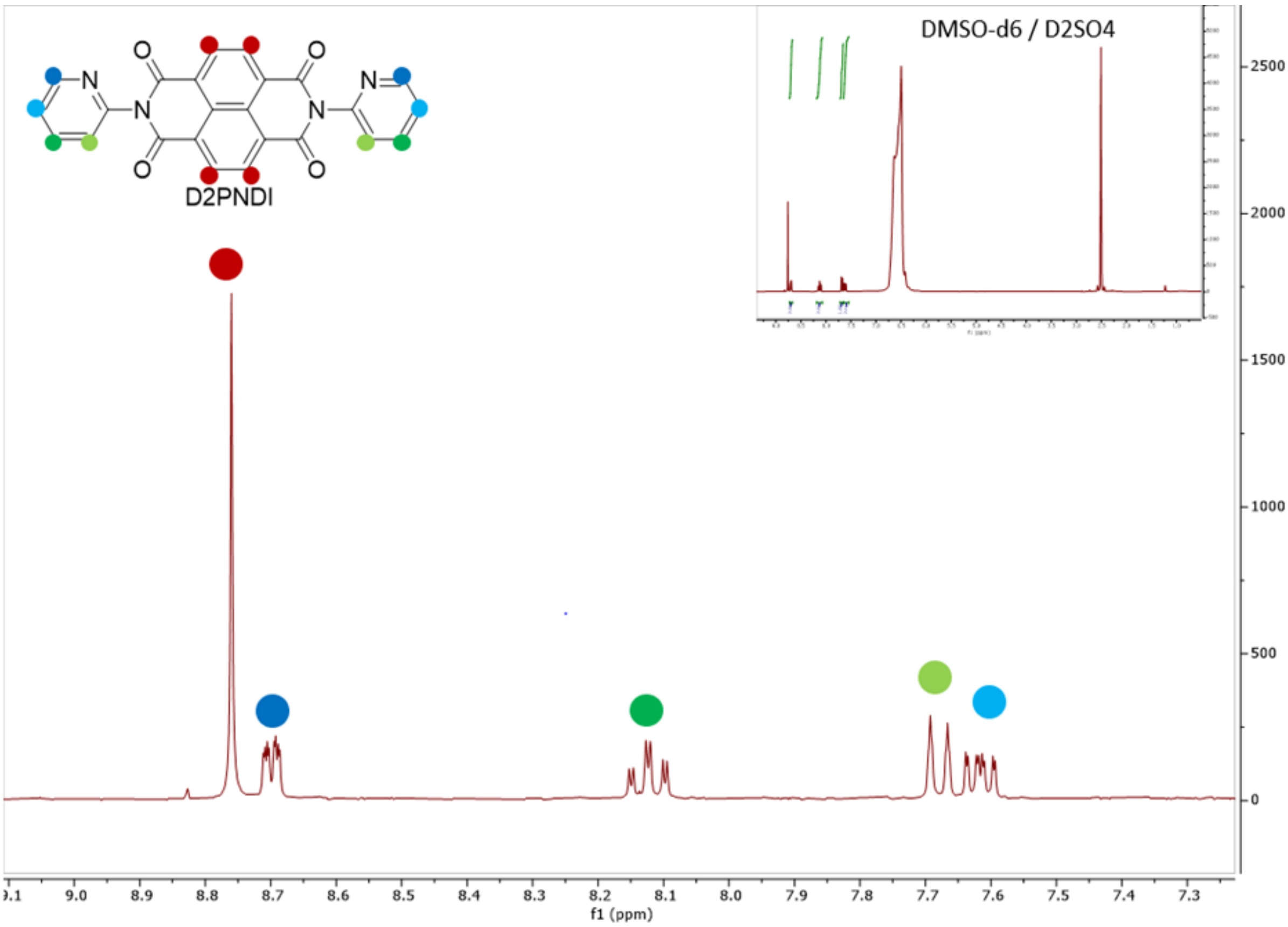

62 % yield, off-white crystalline powder, ^{1}H NMR (300 MHz, DMSO-d6, D_2SO_4) δ 8.76 (s, 4H), 8.69 (8.70 (ddd, J = 4.9, 1.9, 0.8 Hz, 2H) 8.12 (td, J = 7.7, 1.9 Hz, 2H), 7.68 (d, J = 7.9 Hz, 2H), 7.62 (ddd, J = 7.5, 4.9, 1.0 Hz, 2H).

D4PNDI

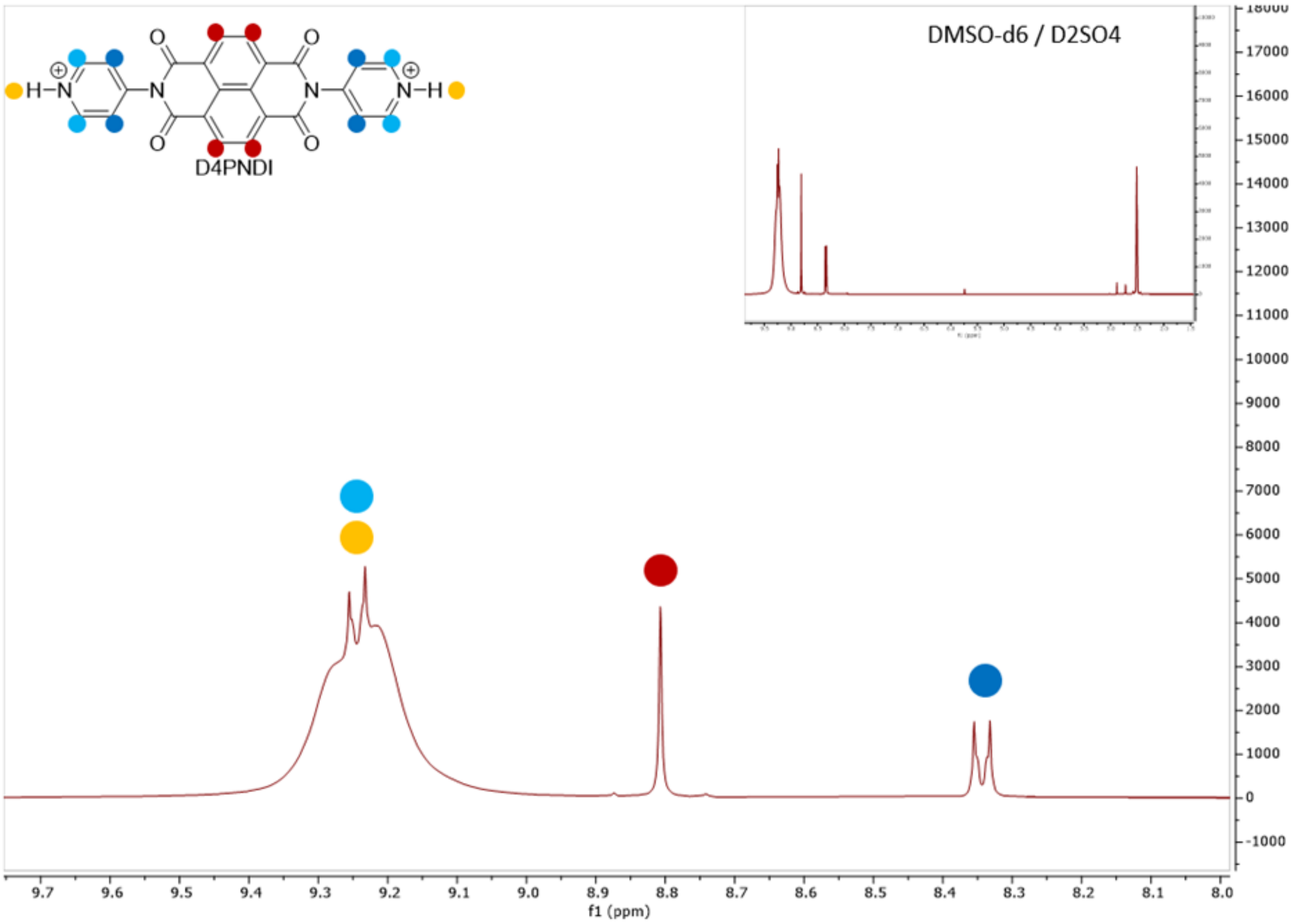

69 % yield, red/orange crystals, ^{1}H NMR (300 MHz, DMSO-d6, D_2SO_4) 9.34 – 9.18 (m, 2H), 9.26 (d, J = 6.6 Hz, 4H), 8.81 (s, 4H), 8.34 (d, J = 7.2 Hz, 4H).

Crystallisation

Co-crystals of pyridyl NDIs and formic acid of sufficient quality for single crystal x-ray diffraction (SCXRD) analysis were produced by dissolving 10 mg of the NDI component in the minimum required volume of glacial formic acid (approx. 2 mL) inside a 20 mL glass vial. The glass vial was tilted to 45°, such that the solution pooled toward one side of the vial. This served to slow solvent evaporation and facilitated continuous solute diffusion from the bulk solution to the nucleating crystals. After 24 hours, the excess formic acid had evaporated, and inside the vial were large, high quality single crystals suitable for x-ray structural analysis.

Single Crystal X-ray Diffraction

The dataset for D4PNDI.2FAH was measured on a Rigaku XtaLAB Synergy diffractometer using a HyPix detector. This data collection was driven and processed and an absorption correction was applied using CrysAlisPro.[S2] The dataset for D2PNDI.3FAH was measured at the Diamond Light Source, Beamline I19-1, using a Dectris PILATUS 2M detector.[S3] This data set was processed and an absorption correction was applied using DIALS 3, XIA2 and AIMLESS.[S4]

Using OLEX2,[S5] the structures were solved using ShelXT,[S6] and were refined by a full-matrix least-squares procedure on F^2 in ShelXL.[S7] All non-hydrogen atoms were refined with anisotropic displacement parameters. Unless otherwise stated, the hydrogen atoms were fixed as riding models and the isotropic thermal parameters (U_{iso}) were based on the U_{eq} of the parent atom.

D4PNDI.2FAH: $C_{26}H_{16}N_4O_8$ (*M* =512.43 g/mol): monoclinic, space group I2/a (no. 15), a = 14.2430(3) Å, b = 6.13440(10) Å, c = 26.1910(5) Å, β = 103.941(2)°, V = 2220.96(8) Å^3, Z = 4, T = 100.00(10) K, μ(Cu Kα) = 0.985 mm^{-1}, *Dcalc* = 1.532 g/cm^3, 11624 reflections measured (6.954° ≤ 2Θ ≤ 158.738°), 2357 unique (R_{int} = 0.0523, R_{sigma} = 0.0407) which were used in all calculations. The final R_1 was 0.0479 (I > 2σ(I)) and wR_2 was 0.1379 (all data).

The structure lies on an inversion centre such that only half the D4PNDI molecule is crystallographically-unique. The hydroxyl hydrogen atom was located in the electron density, and the position and isotropic thermal parameters were freely refined.

D2PNDI.3FAH: $C_{27}H_{18}N_4O_{10}$ (*M* =558.45 g/mol): monoclinic, space group Pc (no. 7), a = 14.68460(10) Å, b = 16.15270(10) Å, c = 20.7724(2) Å, β = 92.2240(10)°, V = 4923.42(7) Å^3, Z = 8, T = 100 K, μ(0.6889 Å) = 0.110 mm^{-1}, *Dcalc* = 1.507 g/cm^3, 99038 reflections measured (2.444° ≤ 2Θ ≤ 71.9°), 43964 unique (R_{int} = 0.0533, R_{sigma} = 0.0872) which were used in all calculations. The final R_1 was 0.1132 (I > 2σ(I)) and wR_2 was 0.3408 (all data).

The asymmetric unit contains four crystallographically independent D2PNDI molecules and twelve formic acid units. The structure occupies a non-centrosymmetric space group, but it was not possible to determine the absolute structure from the diffraction data.

The crystal was severely affected by solvent loss. Despite being mounted in person at the Diamond synchrotron from a microscope located in the hutch, some problems with the data were caused by loss of solvent during transfer from the microscope to the cryostream. This was the best dataset and refinement that could be achieved.

Although all hydrogen atoms were fixed as riding models, AFIX 147 was used for the hydroxyl hydrogen atoms such that the torsion angle was refined. All hydrogen bond requirements have been satisfied.

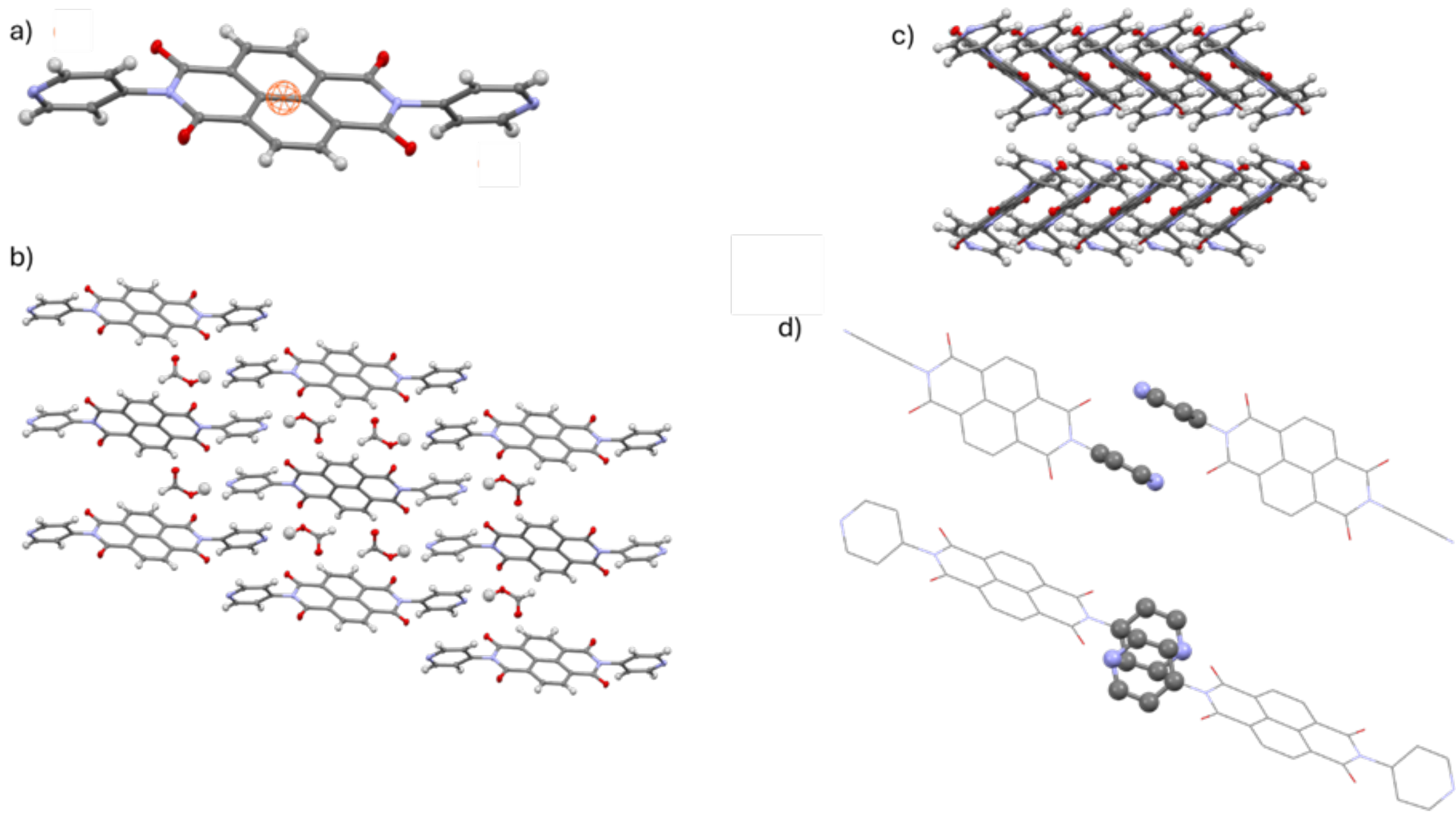

Figure S1 *a) inversion symmetry element at the centre of the NDI core. b) packing view along the b axis showing the FAH containing channels between NDI molecules. c) π-π stacking of NDI cores running along the b axis. d) π-π stacking of pyridyl rings of neighbouring pyridyl NDI molecules with approximate interplanar separation of 3.6 Å.*

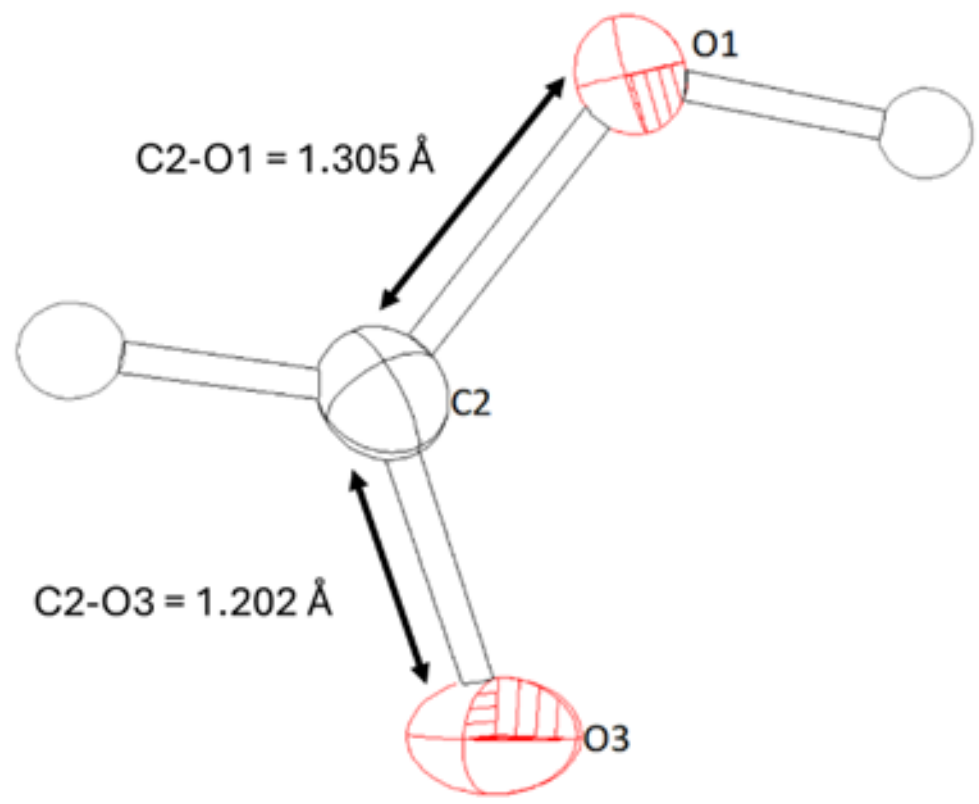

Figure S2 *Disparity between C-O bond lengths in all formic acid species present in D4PNDI·2FAH suggest the presence of an OH group in the formic acid molecule, therefore this structure (in the ground state) is a co-crystal rather than a salt.*

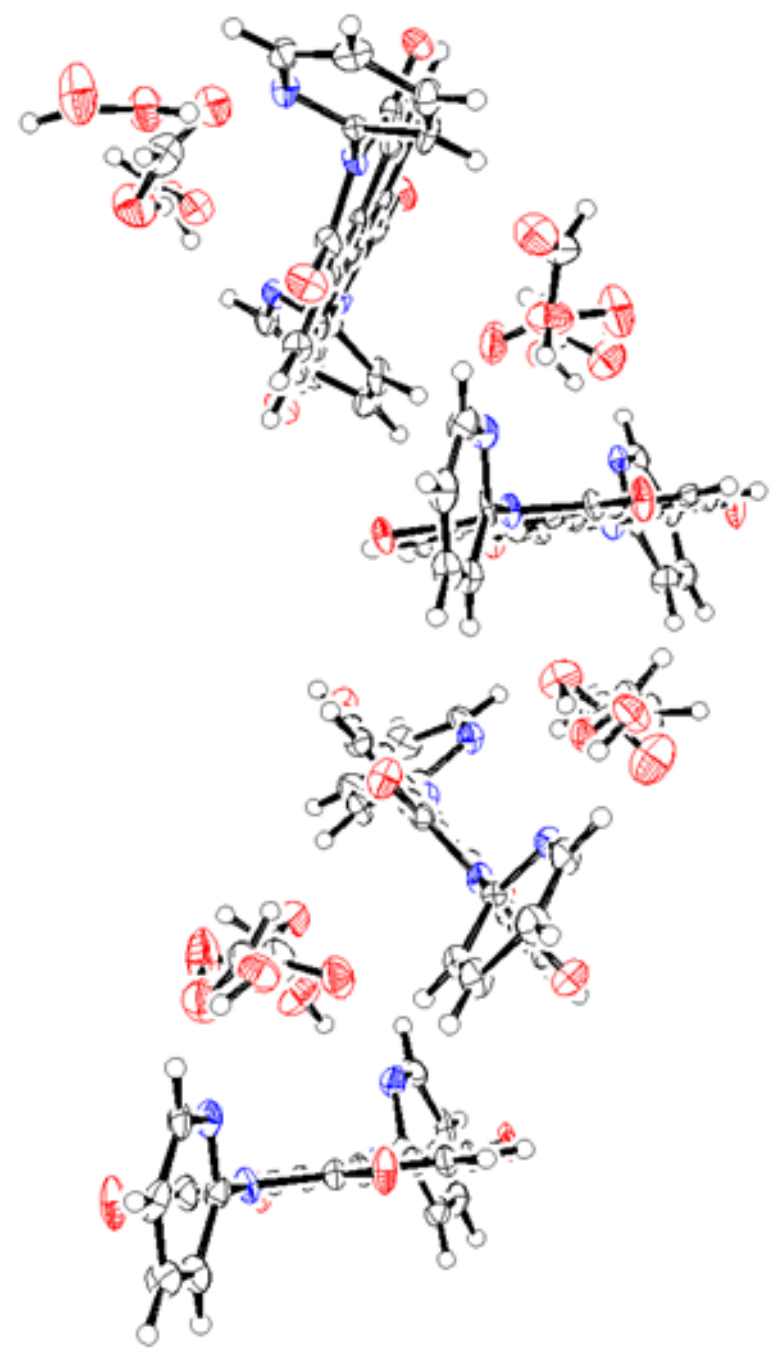

Figure S3 *The unusually large asymmetric unit of D2PNDI·3FAH comprising four pyridyl NDI molecules each associated with a FAH hydrogen-bonded trimer.*

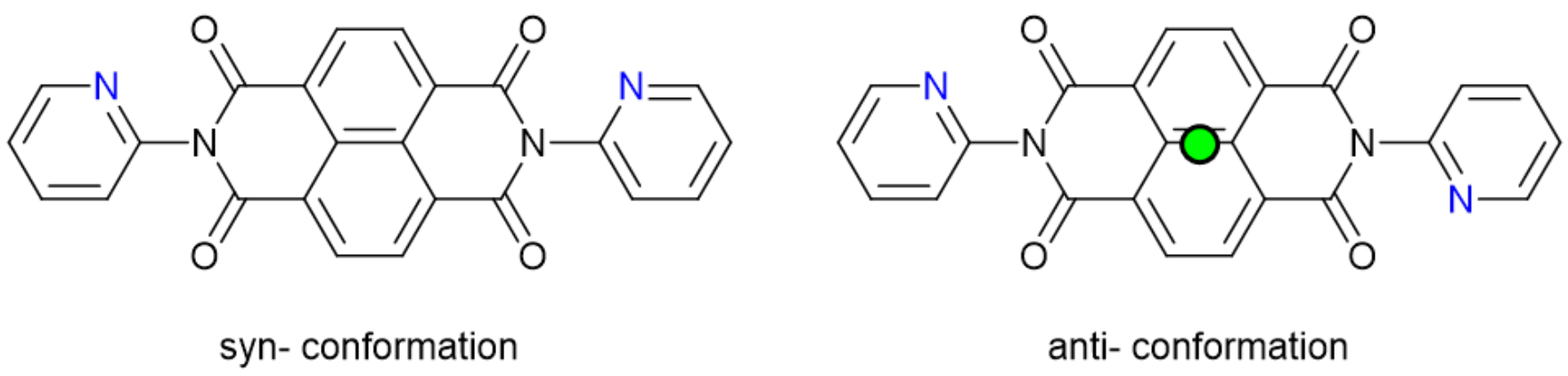

Figure S4 *Comparison between syn- and anti- conformations of D2PNDI. The inversion centre in the anti- conformation is represented by a green circle.*

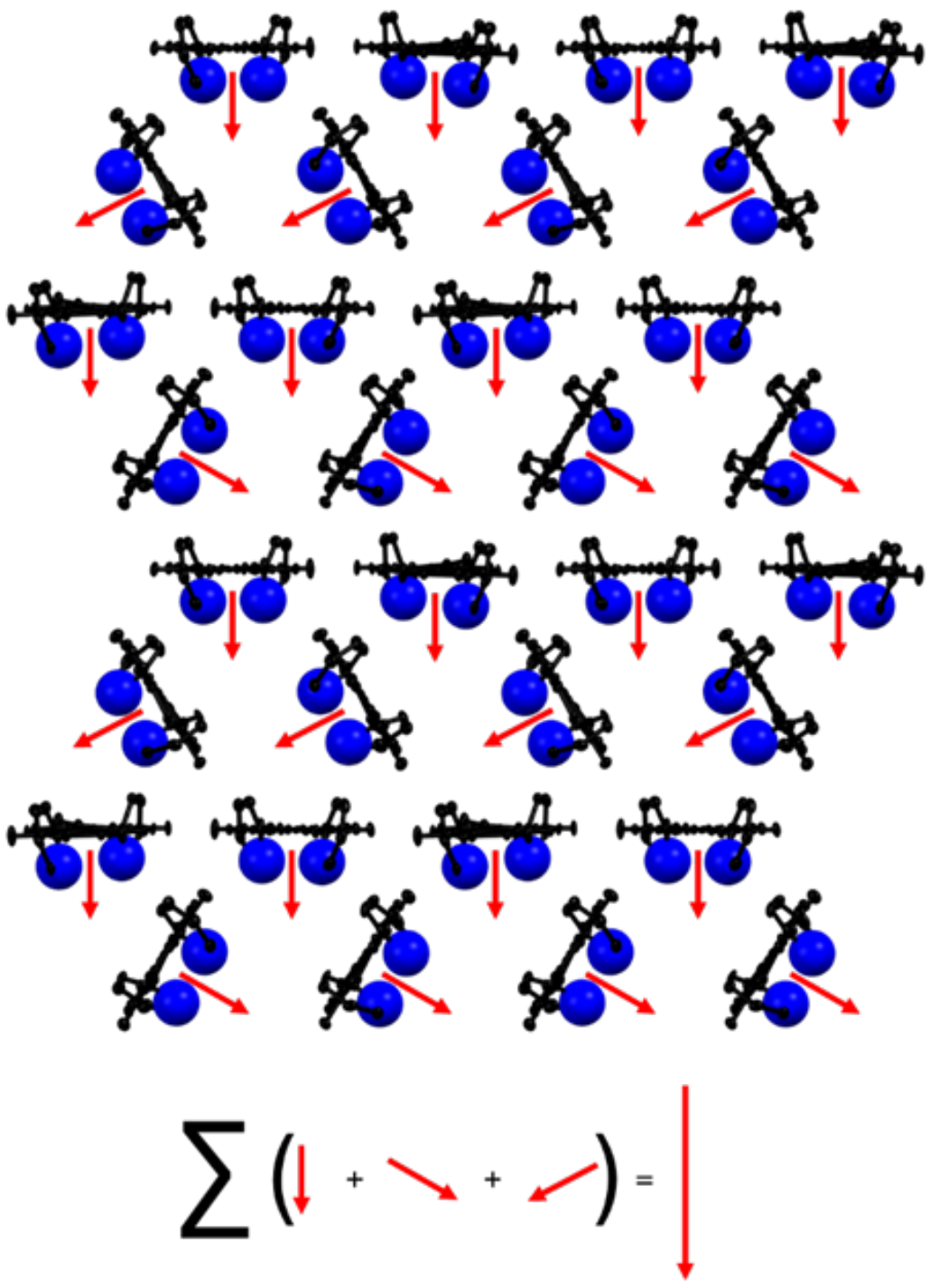

Figure S5 *Qualitative representation of how horizontal dipole moment vectors cancel, while the vertical dipole moment vectors add up, in the crystal structure of D2PNDI·3FAH. Pyridyl nitrogen atoms are highlighted in blue with dipole moments represented by red arrows.*

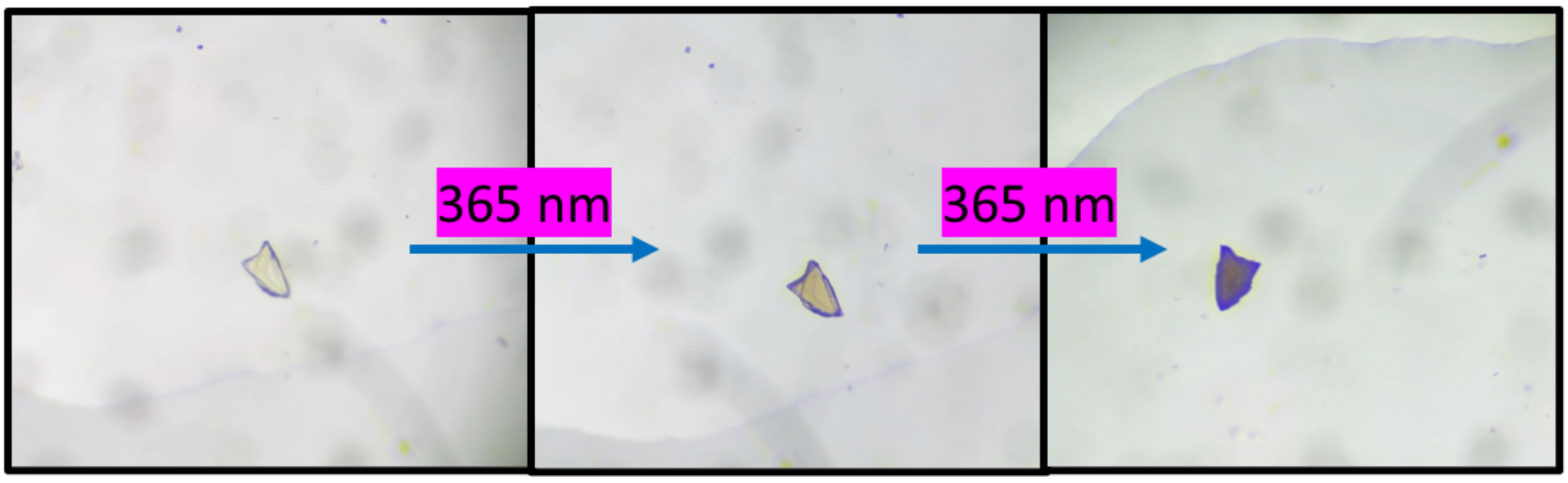

Figure S6 *The crystal shows reversible photochromism upon irradiation by 365 nm UV light.*

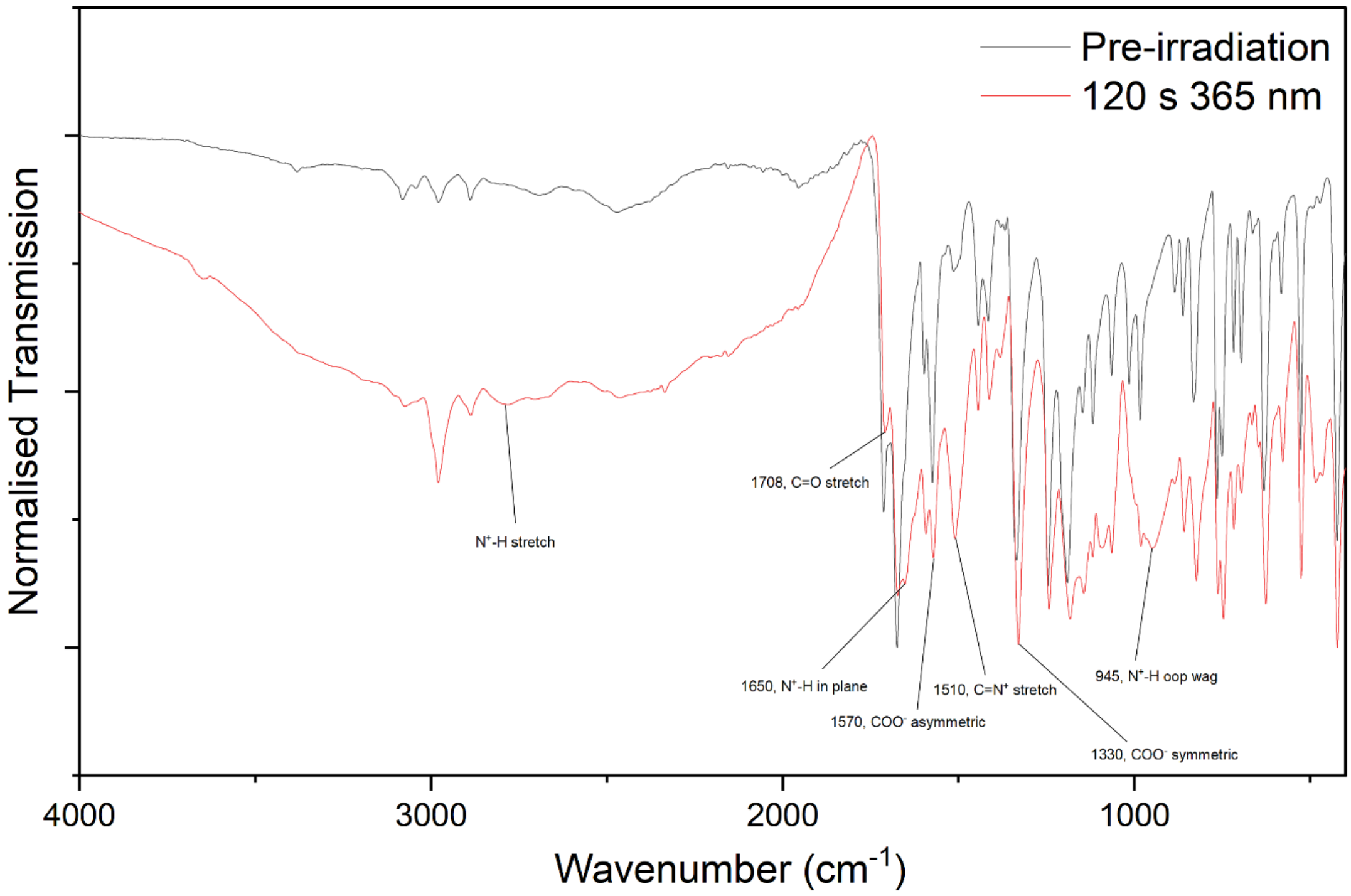

Figure S7 *Overlaid ATR-FTIR spectra of D4PNDI·2FAH before and after irradiation.*

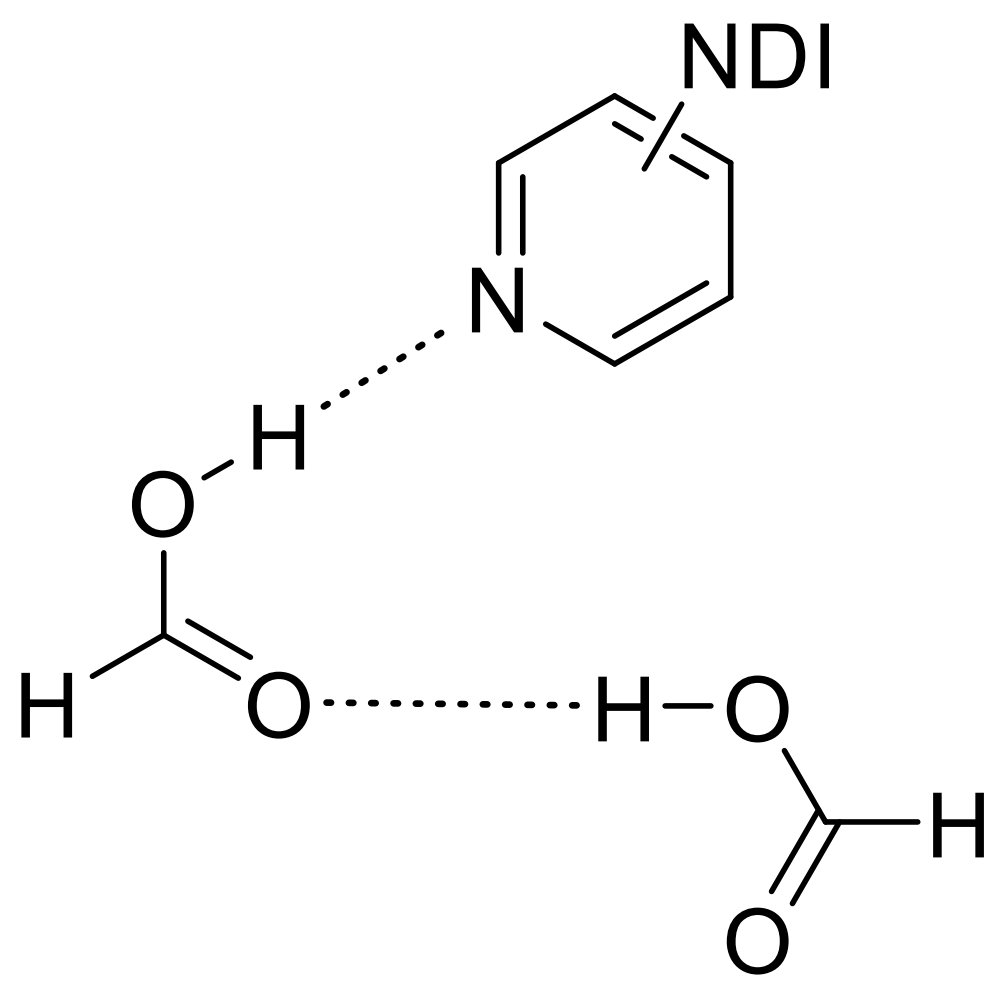

Figure S8 *Additional hydrogen bond in the FA trimer in D2PNDI·3FAH withdraws electron density from the formic acid through the O-H···O contact.*

Diffuse reflectance UV-Vis spectroscopy

Powdered co-crystal samples were irradiated with a 365 nm UV lamp and diffuse reflectance spectra were recorded on a Shimadzu UV-3600i spectrometer. Spectra were recorded at 40 second intervals up to 4000 seconds to monitor the photochromic response and recovery.

Solid-state FTIR spectroscopy

ATR-FTIR spectra were recorded on powdered samples before and after UV irradiation (365 nm, 120 s) using a PerkinElmer spectrum two spectrometer. Spectra were collected between 400 and 4000 cm^{-1}.

References

S1 A. Ishaq, J.O. Ogar, M.M. Mahmud D.E. Schier, S.P. Argent, N.R. Champness, 2026, *submitted*.

S2 CrysAlisPro, Rigaku Oxford Diffraction, 2020 & 2021.

S3 D. R. Allan et al., *Crystals* 2017, *7*(11), 336.

S4 J. Beilsten-Edmands et al., *Acta Cryst*. 2020, D76, 385-399; P. Evans, *Acta Cryst*. 2006, D62, 72-82; P.R. Evans and G.N. Murshudov, *Acta Cryst*. 2013, D69, 1204-1214; M. D. Winn et al., *Acta Cryst*. 2011, D67, 235-242; G. Winter, *J. Appl. Cryst*. 2010, 43, 186-190; G. Winter et al., *Acta Cryst*. 2018, D74, 85-97.

S5 O. V. Dolomanov, L. J. Bourhis, R. J. Gildea, J. A. K. Howard, H. Puschmann, *J. Appl. Crystallogr.* 2009, *42*, 339-341.

S6 G. M. Sheldrick, *Acta Cryst.* 2015, *A71*, 3-8.

S7 G. M. Sheldrick, *Acta Cryst.* 2015, *C71*, 3-8.
